# Ocular ultrasound evaluation of optic nerve sheath diameter in military environments

**DOI:** 10.1186/s40779-019-0207-8

**Published:** 2019-05-25

**Authors:** Maddalena De Bernardo, Livio Vitiello, Palmiro Cornetta, Nicola Rosa

**Affiliations:** Department of Medicine, Surgery and Dentistry, “Scuola Medica Salernitana”, University of Salerno, Via S. Allende – 84081 – Baronissi, Salerno, Italy

**Keywords:** Blooming effect, Intracranial pressure, Optic nerve sheath diameter

## Abstract

In this letter to the Editor, we would like to comment on the article by Betcher et al., concerning the possibility of teaching military trainees to obtain accurate optic nerve sheath diameter measurements, using a brief didactic and a hands-on training session. In particular, this letter notes the importance of the measurement of optic nerve sheath diameter in detecting the eventual elevated intracranial pressure following traumatic brain injury, highlights several limitations in the use of B-scan for such a purpose and suggests a more accurate evaluation with the standardized A-scan.

**Dear Editor,**

In their remarkable article, Betcher et al. [1] evaluated the feasibility of ultrasound measurements of the optic nerve sheath diameter (ONSD) performed by novice ultrasonographers in a military environment to assess the presence of intracranial hypertension.

We congratulate the authors on their interesting paper, but we would like to comment on some aspects of ONSD assessment by ultrasound.

In this study, Betcher et al. properly stated that there are several concerns related to the “narrow window for differentiation between normal and abnormal” in regard to ONSD ultrasound measurements, given that the cut-off values for normal and abnormal ONSD have not been widely accepted. The authors also correctly stated that this method “is prone to error from shadowing by the lamina cribrosa or refraction artifacts related to insonation through the lens”, and the authors tried to overcome this problem by utilizing the axial plane and avoiding the lens.

Unfortunately, this approach could not be a solution because the real problem is related to the ultrasound technique.

Ultrasonography has been utilized in the ophthalmological field since the late 1970s to diagnose and measure ocular and orbital structures [2, 3]. Over the years, it has been clearly shown that B-scan ultrasonography, or B-scan, is very useful for detecting eventual lesions, whereas amplitude scan ultrasound biometry, or A-scan, is not only suitable for detecting and diagnosing ocular and orbital lesions but also much more reliable in making the measurements because it is not influenced by the so-called blooming effect that is present with B-scan [4–8].

Due to this effect, which is related to the absence of a standard sensitivity setting, a lesion that is measured utilizing different gains will appear larger as the gain decreases, and smaller as the gain increases.

For this reason, B-scan measurements of the ONSD, influenced by the blooming effect, will be altered in a significant way.

Due to the aforementioned limits, we would like to suggest using a standardized A-scan to produce more exact and objective measurements when appraising intracranial pressure because this technique shows easily discernible high reflective spikes from the interface between the arachnoid and subarachnoidal fluid, and it is also free from the blooming effect. For this reason, the A-scan also provides more accurate reference range values that can be utilized worldwide [9, 10]. We are also aware that most point-of-care ultrasound devices in a military setting do not have the ability to perform an A-scan, but we believe that the poor reliability of B-scan can yield incorrect results that can be worse than having no results.

Moreover, we would like to highlight that an increase in ONSD does not conclusively prove the presence of increased intracranial pressure, but it can also be caused by other diseases, such as optic nerve meningioma or optic neuritis. This ambiguity could be overcome by utilizing the A-scan “30 degree test,” which consists of a measurement of the optic nerve performed with the patient looking straight ahead, and then to the lateral side. A test result showing a decrease in the maximal diameter of at least 5% will prove the presence of intracranial hypertension caused by increased subarachnoidal fluid that produces distension of the ONSD [11–13].

Furthermore, we would like to note how the probe should be used to obtain more trustworthy measurements. As we cannot exactly define direction of the gaze of a patient with closed eyes, during the ultrasound evaluation in ophthalmology, the B- or A-scan probe is usually utilized with open lids, using methylcellulose and anesthetic drops [14–16]. This approach allows the eye position to be unmistakably visualized, making the probe orientation much more reliable and avoiding mistakes in detecting gaze direction [17].

Finally, we would like to stress the necessity of expertise and superior knowledge of orbital and ocular anatomy to yield reproducible and reliable measurements. In fact, the acceptable performance of novice ultrasonographers is a concern despite comparisons of their measurements with those of trained physicians, and this performance should be validated by further studies utilizing larger sample sizes and trainees with the standard level of training needed for ultrasonographic eligibility. The minimum training requirements in the physics of ultrasonography normally require more time and effort [18, 19].

## Abbreviation

ONSD: optic nerve sheath diameter
